# Infectious endocarditis caused by *Helcococcus kunzii* in a vascular patient: a case report and literature review

**DOI:** 10.1186/s12879-015-0984-y

**Published:** 2015-06-23

**Authors:** Romain Lotte, Laurène Lotte, Nicolas Degand, Alice Gaudart, Sylvie Gabriel, Mouna Ben H’dech, Mathilde Blois, Jean-Paul Rinaldi, Raymond Ruimy

**Affiliations:** Department of Bacteriology at Nice Academic Hospital, Nice, France; Department of Medical Biology, Monaco Princess Grace General Hospital, Monaco, Monaco; Department of Cardiology, Monaco Princess Grace General Hospital, Monaco, Monaco; Nice Medical University, Nice-Sophia Antipolis University, Nice, France

## Abstract

**Background:**

*Helcococcus kunzii* is a facultative anaerobic bacterium that was first described by Collins et al. in 1993, and was initially considered as a commensal of the human skin, in particular of lower extremities. Human infections caused by *H. kunzii* remain rare with only a few cases published in the pubmed database. Nevertheless recent reports indicate that this microorganism has to be considered as an opportunistic pathogen that can be involved in severe infections in human. To the best of our knowledge, we describe here the first known case of infectious endocarditis caused by *H. kunzii.*

**Case presentation:**

A 79 year-old man reporting severe polyvascular medical history attended the emergency ward for rapid deterioration of his general state of health. After physical examination and paraclinical investigations, the diagnosis of infectious endocarditis on native mitral valve caused by *Helcococcus kunzii* was established based on Dukes criteria. MALDI-TOF mass spectrometry and 16S rDNA sequencing allowed an accurate identification to the species level of *Helcococcus kunzii.* The patient was successfully treated by a medico-surgical approach. The treatment consisted in intravenous amoxicillin during four weeks and mitral valve replacement with a bioprosthestic valve. After an in depth review of patient’s medical file, the origin of infection remained unknown. However, a cutaneous portal of entry cannot be excluded as the patient and his General Practitioner reported chronic ulcerations of both feet.

**Conclusions:**

We describe here the first case of endocarditis caused by *H. kunzii* in an elderly patient with polyvascular disease. This report along with previous data found in the literature emphasizes the invasive potential of this bacterial species as an opportunistic pathogen, in particular for patient with polyvascular diseases. MALDI-TOF mass spectrometry and 16S rDNA sequencing are reliable tools for *H. kunzii* identification. We also sequenced in this work *H.kunzii* type strain 103932T CIP and deposited in the Genbank under accession number KM403387. We noticed a 14 base difference between our sequence and the original sequence deposited by Collins et al. under Genbank accession number X69837. Hopefully, the spread of next generation sequencing tools would lead to a more accurate classification of clinical strains.

## Background

*Helcococcus kunzii* is a facultative anaerobic bacterium that was first described by Collins et al. in 1993, and was initially considered as a commensal of the human skin [1–3]. Recent reports of invasive infections caused by *H. kunzii* indicate that this microorganism has to be considered as an opportunistic pathogen that can be involved in severe infections in human [4–6]. We describe here the first known case of infectious endocarditis caused by *H. kunzii* in a patient with polyvascular disease and further provide a short review of the literature on infections related to *H. kunzii*.

## Case Presentation

A 79 year-old man attended the emergency ward in February 2014 for chills, diarrheas, and a rapid deterioration of his general state of health. The patient was a former heavy smoker and also suffered from alcoholism, dyslipidemia and high blood pressure. He had no history of drug use. Noteworthy medical history consisted in ischemic heart disease treated by coronary angioplasty in 2002, left and right carotid surgery by endarterectomy successively in 2004 and 2008, and a vascular stenting of an abdominal aortic aneurysm in 2010.

On admission, hemodynamic status was stable. The pulse rate was 81 beats/min and respiratory rate was normal. Body temperature was 38.5 °C. Interestingly, the auscultation showed light bilateral crackles in the lower third of lungs, a systolic mitral murmur grade III/IV and no carotid murmur. No sign of thrombophlebitis of the lower limbs was found. Laboratory investigations revealed inflammatory markers such as elevated C-reactive protein (114 mg/L), leukocytosis (18 × 10^9^/L) and normocytic anaemia (12 g/dL). Considering the clinical and laboratory investigations, infectious endocarditis (IE) was suspected and the patient was promptly transferred to the department of cardiology.

**Fig. 1:**
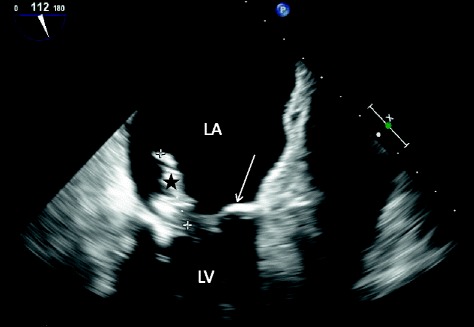
Transesophageal echocardiogram of infected native mitral valve. Echocardiography visualized a 28.6 mm multilobulated vegetation (black star), attached to the native mitral valve (arrow). Left atrium (LA). Left ventricle (LV). The 3D color Doppler (not shown) revealed a severe valve dysfunction with mitral regurgitation

Transesophageal echocardiography (TEE) visualized a 28.6 mm vegetation on the mitral valve (Fig. 1), and a 3D color Doppler showed a severe valve dysfunction with mitral regurgitation, which was consistent with mitral IE on native valve. A complete imaging workup, including spinal cord and brain magnetic resonance imaging, did not detect any other septic location. Of note, abdominal computed tomography did not show any perigraft fluid or other evidence of suspicion of an infected aortic vascular graft. The ocular examination of the patient was normal. Three sets of blood cultures were successively drawn (one set every 12 h) on the day of admission and the day after. Blood cultures were processed with a Bactalert 3D system (BioMérieux, France). Four out of six blood culture bottles (3 anaerobic and 1 aerobic) grew respectively in 7, 8, 24 and 72 h. The remaining aerobic bottles were negative after prolonged incubation (up to 28 days). Gram staining directly performed on the positive blood cultures yielded Gram-positive cocci arranged in clumps. Cultures grew aerobically and anaerobically on Columbia agar plates supplemented with 5 % sheep blood (BioMérieux, Marcy l’Etoile, France) after 24 h of incubation as non-hemolytic, grey, pinpoint colonies. Catalase and oxidase reactions were negative. Phenotypic characterization using API system (BioMérieux, API 20 Strep) did not provide any reliable identification (*Aerococcus viridans* with a doubtful significance, numerical profile: 4100413). In order to achieve an acute bacterial identification the strain was sent to our laboratory (Nice University Hospital). Matrix-assisted laser desorption/ionization time of flight (MALDI-TOF) mass spectrometry using Microflex LT with Biotyper v2.3 database (Bruker Daltonics, Bremen, Germany) directly on colonies identified *Helcococcus kunzii* (log score value of 2.38 matching with *H. kunzii* type strain CIP 103932T). Identification was confirmed by 16S rDNA gene sequencing on colonies using forward (A2: 5’AGAGTTTGATCATGGCTCAG3’) and reverse (S15: 5’GGGCGGTGTGTACAAGGCC3’) primers as previously described [7]. Blast analysis of the partial 16S rDNA sequence of our strain (1346 nucleotides, deposited in Genebank under accession number KM403388) showed 98.9 % identity (14 nucleotides differences) with 16S rDNA sequence of *H. kunzii* CIP 103932T deposited in Genbank under accession number X69837 by Collins et al. in 1993 when they first described this species [1]. Antimicrobial susceptibility testing (AST) was performed using the E-test method on Mueller-Hinton agar supplemented with 5 % sheep blood (BioMérieux, Marcy l’Etoile, France) incubated at 36 °C in 5 % CO_2_ for 24 h according to the Clinical and Laboratory Standards Institute (CLSI) interpretative standard for *Streptococcus* species [8]. The strain was highly susceptible to all β-lactams tested. It was also susceptible to clindamycin, and vancomycin, but had reduced susceptibility to gentamicin. The MICs (μg/L) of drugs for this strain are reported in Table 1. The patient was initially treated empirically with intra-venous amoxicillin (100 mg/kg per day) and gentamicin adapted to the renal function. Once the results of the blood cultures were available, the treatment was followed by intra-venous amoxicillin monotherapy (100 mg/kg per day) for a total duration of 4 weeks. 5 days after admission, the patient underwent a mitral valve replacement with a bioprosthestic valve. Vegetation length (>15 mm) is a strong predictor of new embolic-event and constituted therefore a major indication for mitral valve surgery in this patient according to the guidelines of the European society of cardiology [9]. Bacteriological analysis of the mitral valve was negative after 5 days of incubation but there was not enough valve tissue remaining for *16S rDNA* PCR analysis. A total of three blood culture sets at days 5 and 6 after admission remained negative despite prolonged incubation up to 4 weeks. Control echocardiography 3 weeks after surgery resulted normal. Moreover, biological and clinical follow-up at 3 and 6 months confirmed a full-sustained cardiac recovery. At 6 months, the complete physical examination only retrieved right hallux ulceration for which microbiological analysis only detected few colonies of *Klebsiella pneumoniae* and *Staphylococcus epidermidis*, but remained negative for anaerobic bacteria.

**Table 1:** Antimicrobial susceptibilities of *Helcococcus kunzii* determined with the E-test method and clinical categorization

Antimicrobial agent	MIC (μg/mL)	Clinical categorization
Penicillin G	<0.016	S
Amoxicillin	<0.016	S
Amoxicillin + clavulanic acid	<0,016	S
Cefotaxime	<0,016	S
Clindamycin	0.032	S
Gentamicin	1,5	I
Vancomycin	0,38	S

## Discussion

The first description of the genus *Helcococcus* was made by Collins et al. in 1993, based on *16S rDNA* sequencing and phylogenetic analysis of a collection of 9 strains of *Aerococcus*-like organisms isolated from human clinical sources [1]. Within this genus, *H. kunzii* was the first species to be characterized (type strain NCFB 2900/CIP 103932T) [1] followed by the species: *Helcococcus pyogenes, Helcococcus sueciensis*, *Helcococcus ovis* [10] and recently *Helcococcus seattlensis* [11]. Briefly, *H. kunzii* is a catalase-negative, facultative anaerobic, non-motile Gram-positive coccus whose cells are arranged in pairs and clusters. The organism grows slowly, producing pinpoint translucent to greyish, non-haemolytic or slightly alpha-haemolytic colonies after 24 h incubation on blood agar, with no difference of growth under 5 % CO_2_ or anaerobic conditions [1, 2]. Biochemical methods are not reliable for *H. kunzii* identification, as assessed by several reports mentioning misidentification of *H. kunzii* as *Aerococcus sp.* or *Aerococcus viridans* [1, 3–5, 12, 13]. In this particular case, phenotypic characterization using API system (BioMérieux, API 20 Strep) misidentified the strain as *Aerococcus viridans* with a doubtful significance (numerical profile: 4100413) as previously described [1, 3–5, 12, 13]. MALDI TOF Mass spectrometry allowed an accurate species level identification of our strain (log score value of 2.38 matching with *H. kunzii* type strain CIP 103932 T). This point can be explained by the expanding MALDI-TOF databases which enable this technique to identify such unfrequently encountered organisms. *16S rDNA* sequencing confirmed the species level identification obtained by MALDI-TOF mass spectrometry. In this work we further performed *16S rDNA* sequencing on the type strain CIP 103932T using primers previously described [7] and we deposited it in the Gene Bank under accession number KM403387. Nucleotide sequence alignment with BioEdit (http://www.mbio.ncsu.edu/BioEdit/) between type strain sequence accession number KM403387 and *16S rDNA* sequence of our *H. kunzii* clinical strain showed there were 0 (0 %) base difference. Interestingly, when performing sequence alignment with BioEdit between our sequence of *H. kunzii* type strain (accession number KM403387) and the Gene Bank deposited *H. kunzii* type strain (sequence X69837) first described by Collins et al. [1] we found a 14 (1,05 %) base difference. The manual sequencing method used by Collins et al. when they first described *H. kunzii* [14] could explain this 14 base pair difference between the two sequences. Hopefully, the spread of new genomic tools such as whole-genome sequencing will help to minimize this type of error that are persisting in bacterial 16S bank databases and will also lead to a an accurate classification of bacterial species based on the whole genome sequence.

Concerning the clinical features, several authors already reported data concerning *H. kunzii*. This bacterium was first considered to be part of the normal flora of the human skin, in particular of lower extremities [2, 3]. More recently, it has been shown to be involved in human infections either in mixed bacterial cultures such as diabetic foot infection [15], plantar phlegmon [16], chronic osteomyelitis of the tibia [17], or severe monobacterial infections such as prosthetic joint infection [4], implantable cardiac device infection [6], abscesses [11–13, 18] empyema, bacteraemia [5] and central nervous system infection [19]. The main clinical and microbiological features of these infections are shown in Table 2. To the best of our knowledge, we report here the first known case of IE caused by *H. kunzii* in an elderly patient with polyvascular disease. According to the modified Duke Criteria for a definitive diagnosis of IE [20], the patient met one major criterion (new valvular regurgitation murmur and positive echocardiogram) and three minor criteria (fever, predisposing factor of heart condition and 3 blood cultures growing an organism that does not commonly cause endocarditis). Interestingly, the origin of infection remains unknown and was not assessed by any microbiological data. On admission, urine sample was positive with *E.coli* 10^3^ CFU/mL. No other microbiological sample was sent to the laboratory for analysis. After an in depth review of the patient’s medical file, it seems that he had no known intravenous drug use history. The patient was alcoholic and ex-smoker. He did not report any recent dental extraction before admission. The literature review suggests that *H. kunzii* is mostly involved in skin infection [11–13, 15, 16, 18] and therefore cutaneous portal of entry cannot be excluded as the patient and his General Practitioner reported chronic ulcerations of both feet. Finally the AST performed on the strain confirmed the high susceptibility of *H. kunzii* to β-lactams previously demonstrated by several authors [2, 4–6, 11–13, 15, 16, 18, 19]. Further more, *H. kunzii* strains often displayed reduced susceptibility to aminoglycosides either gentamicin or amikacin [4, 11, 18, 19]. This could have an impact on antimicrobial therapy as gentamicin is frequently prescribed in combination with beta-lactams for empirical coverage of IE. In our patient, a medico-surgical approach consisting in mitral valve replacement and combination of intravenous amoxicillin and gentamicin followed by amoxicillin monotherapy led to a full-sustained cardiac recovery. Altogether, considering the well-established synergistic activity of the β-lactam-aminoglycoside association, such combination might be used in case of IE caused by *H. kunzii*, even if the isolate displays reduced susceptibility to aminoglycosides. Nevertheless, further studies will be required to better characterize the antimicrobial susceptibility pattern of this newly recognized emerging pathogen.

**Table 2:** Main features of reported cases of *Helcococcus kunzii* infections

Sex/age of patient (years)	Underlying condition(s)	Type of infection	Treatment	Outcome	Other bacteria	Methods of identification	Author
M/41	IV-drugs user	Bacteraemia	3 weeks of penicillin G and cloxacillin IV	Recovery	None	-API 20 Strep system, (bioMérieux, Marcy l’Etoile, France), (code 4100413)-16S rDNA gene sequencing	Woo et al. 2005 [5]
M/83	Hypertension, diabetes, prostate cancer	Brain abscess	2 weeks of ceftriaxone and metronidazole IV, then oral amoxicillin-clavulanic acid and then ceftriaxone and metronidazole and vancomycin IV. Total duration of antimicrobial therapy 12 weeks.	Recovery	None	-Vitek2 system, (bioMérieux, Marcy l’Etoile, France) -Matrix-assisted laser desorption time-of-flight mass spectrometry (MALDI-TOF MS; Vitek MS bioMérieux) -16S rDNA gene sequencing	Sridhar et al. 2014 [19]
M/55	Smoker, alcoholic and IV-drugs user	Empyema thoracic	8 weeks of amoxicillin-clavulanic acid	Recovery	None	-API 20 Strep system, (code 4100413) -16S rDNA gene sequencing	Woo et al.2005 [5]
M/39	Osteochondritis	Prosthetic joint chronic infection	Clindamycin and gentamicin	Recovery	None	-API 20 Strep system,(code 4100413) -16S rDNA gene sequencing	Perez-Jorge et al. 2011 [4]
M/75	NA	Infection of implantable cardiac device	Association of flucloxacilline (2 g × 4/day) and benzylpenicllin (2.4 × 4/day) IV then association of vancomycin and clindamycin (14 days) IV and then association of oral amoxicillin and rifampicin for four weeks	Recovery	None	-BBL Crystal^TM^ System (Baltimore, MD, USA)-BD Phoenix ^TM^ Automated Microbiology System (Baltimore, MD, USA) -16S rDNA gene sequencing	Mc Nicholas et al. 2011 [6]
W/57	None	Breast abscess	Oral cephalexin (0.5 g/day) for 5 days	Recovery	None	-API 20S Strep system, (code 4100413),-16S rDNA gene sequencing	Chagla et al. 1998 [13]
W/36	None	Post chirurgical foot abscess	Pristinamycine and rifampicin	Recovery	None	-Rapid ID 32 Strep system (bioMérieux, Marcy l’Etoile, France), (*Aerococcus viridans*) -16S rDNA gene sequencing	Riegel et al. 2003 [18]
M/36	High blood pressure, obesity and hypercholesterolemia	Sebaceous cyst infection associated with cellulitis	Flucloxacillin (1 g × 4/day) IV and then oral flucloxacillin (0.5 g × 4/day) for 5 days	Recovery	None	-API 20 Strep system, (code 4100413)	Peel et al. 1997 [12]
M/86	Malignant melanoma and congenital thrombocytopenia	Osteomyelitis	Cefuroxime (3 × 1.5 g/day) and metronidazole (2 × 500 mg/day) and then amoxicillin-clavulanic acid (2 × 1 g/day) for 6 weeks PO	Recovery	Anaerobic bacteria and germs of normal skin flora	Not available	Stanger et al. 2013 [17]
M/68	Coronary artery disease and colonic polyps	Inner thigh wound from trauma	Vancomycin and piperacillin-tazobactam inpatient and cephalexin outpatient	NA	*Staphylococcus aureus*	-API 20 Strep system and VITEK 2 (bioMérieux, Marcy l’Etoile, France),	Chow et al. 2014 [11]
						-16S rDNA gene sequencing	
M/25	Post-traumatic stress disorder	Toe abscess	Sulfamethoxazole, cephalexin	NA	*Staphylococcus aureus*	-API 20 Strep system and VITEK 2,	Chow et al. 2014 [11]
						-16S rDNA gene sequencing	
M/79	Hypertension and severe intermittent claudication of both legs	Plantar phlegmon	Amoxicillin-clavulanic acid (3 weeks), surgical debridement and incision and drainage of the phlegmon	NA	*Klebsiella oxytoca, Bacteroides fragilis*	-VITEK 2 GP card identification system (bioMérieux, Marcy l’Etoile, France) -16S rDNA gene sequencing	Lemaître et al. 2008[16]
M/58	Diabetes and end-stage renal disease	Ulcerative lesion of foot	3 weeks of piperacillin/tazobactam IV	Recovery	*Proteus mirabilis*	-Matrix-assisted laser desorption time-of-flight mass spectrometry (MALDI-TOF MS; Bruker GmbH, Bremen, Germany)	Park et al. 2014 [15]
						-Vitek2 GP system (bio-Mérieux, Marcy l’Etoile, France)	
						-16S rDNA gene sequencing	

## Conclusions

We describe here the first case of endocarditis caused by *H. kunzii* in an elderly patient with polyvascular disease. Accurate identification of this uncommon species allowed by MALDI-TOF mass spectrometry emphasizes the growing importance of this technique in routine microbiological diagnosis. Interestingly, this report along with the previous cases of invasive infections caused by *H. kunzii* indicate that this recently discovered bacterium has to be considered as a true opportunistic emerging pathogen and not only as a commensal of the human skin as initially thought in its first description. *16S rDNA* sequencing confirmed the bacterial identification and allowed us to detect differences between two sequences of the same *H. kunzii* type strain (gene Bank accession numbers KM403387 and X69837) which could be explained by the sequencing methods performed and will have to be investigated by whole sequence analysis.

### Consent

Written informed consent was obtained from the patient for publication of this case report and any accompanying images. A copy of the written consent is available for review by the Editor-in-Chief of this journal.

## Abbreviations

IE: Infectious endocarditis
TEE: Transesophageal echocardiography
